# On the Simulation-Based Reliability of Complex Emergency Logistics Networks in Post-Accident Rescues

**DOI:** 10.3390/ijerph15010079

**Published:** 2018-01-06

**Authors:** Wei Wang, Li Huang, Xuedong Liang

**Affiliations:** 1 College of Harbor, Coastal and Offshore Engineering, Hohai University, Nanjing 210098, China; 13813826667@hhu.edu.cn; 2 School of Public Administration, Hohai University, Nanjing 210098, China; lily8214@hhu.edu.cn; 3 Business School, Sichuan University, Chengdu 610065, China

**Keywords:** complex network, emergency logistics network, reliability, simulation analysis of attacks

## Abstract

This paper investigates the reliability of complex emergency logistics networks, as reliability is crucial to reducing environmental and public health losses in post-accident emergency rescues. Such networks’ statistical characteristics are analyzed first. After the connected reliability and evaluation indices for complex emergency logistics networks are effectively defined, simulation analyses of network reliability are conducted under two different attack modes using a particular emergency logistics network as an example. The simulation analyses obtain the varying trends in emergency supply times and the ratio of effective nodes and validates the effects of network characteristics and different types of attacks on network reliability. The results demonstrate that this emergency logistics network is both a small-world and a scale-free network. When facing random attacks, the emergency logistics network steadily changes, whereas it is very fragile when facing selective attacks. Therefore, special attention should be paid to the protection of supply nodes and nodes with high connectivity. The simulation method provides a new tool for studying emergency logistics networks and a reference for similar studies.

## 1. Introduction

With the rapid development of global commerce, accidents such as diseases or public health emergency frequently occur, resulting in numerous casualties and heavy economic losses. Emergency logistics provide material support for accidents, which makes a reliable emergency logistics network vital [1]. A complex emergency logistics network is a type of temporal and spatial evolving dynamic network that involves a wide variety of unstructured data. An effective emergency logistics network is helpful for improving the performance of emergency rescue and post-accident operations in sustainability. As a typical complex network, an emergency logistics network may be the victim of attacks and damages arising from uncertainties, such as randomness, diffusivity, and the aftermath of an outbreak of emergencies. These issues may cause local failure or paralysis of the network, which would introduce serious aftereffects into the entire socio-economic system. Thus, this topic has garnered the attention of emergency personnel. When an emergency logistics network is under sudden attack, it is vital to swiftly return it to a sound working mode, and reliability is the key to accomplishing this task. Reliability analysis is an indispensable process in emergency network planning, design, and management. Therefore, increasing emergency material reserves and optimizing the reliability of emergency logistics networks are key factors in emergency management systems. Therefore, we must further the research on the reliability of networks to relieve the effects of network emergencies. 

Many researchers have conducted studies on emergency networks [2,3,4,5,6,7]. For instance, Carter put forward the early connotation of emergency logistics [2]. Guan et al. combined the hub-spoke network model with the coverage model for site selection of emergency facilities [4]. Razi et al. constructed a multi-objective incident-based boat allocation model to allocate search and rescue resources [5]. Jin et al. proposed a set of operation scheduling schemes for marine oil spill emergency vessels [6]. Nolz et al. studied the risk of discontinued transportation paths due to damaged infrastructure and addressed such risk as a multi-objective optimization problem [7].

Emergency logistics networks involve a high degree of uncertainty [8]. In view of the uncertain demand and logistics networks in emergency relief, researchers have conducted studies on emergency networks under uncertainty based on integer programming [9], stochastic programming [10], fuzzy theory [11,12], and robust optimization [13,14,15,16,17]. For example, Wagner et al. developed deterministic and stochastic models that optimally match supplies of various types of boats to station demands [9]. Ai et al. studied the location-allocation problem of water emergency supplies under triangular fuzzy demand [12].

Researchers have recently paid more attention to emergency network reliability. For instance, Hong et al. conducted research on emergency logistics using simulations [18]. Masoud et al. calculated the supply chain reliability of humanitarian supply transportation; they established a mathematical model for the routing and scheduling of humanitarian supplies and assessed the reliability of various designs [19].

It is very difficult to discover a universal pattern for the connected state of a network using conventional methods under the circumstance of a partial network failure. Therefore, complex network theory, which has pinpointed the faults of the North American power grid, provides a new method for research into emergency networks. Existing research results show that a logistics network has the nature of a “small-world network” and a “scale-free network”, and complex network theory can be used in the study of a logistics network [20,21]. To date, several researchers have conducted studies using complex network theory. For example, Carlos et al. studied the characteristics of logistics networks by applying this theory and discussed the rationality of its application to general cargo and containership routes [22]. Ehsan et al. used the multi-agent model and evolutionary computation to disseminate public advisories about hazardous material emergencies [23]. However, generally, these studies represent only the beginning of emergency logistics network research using complex network theory; there is little optimization technology research based on reliability theory [24,25]. Edrissi et al. found critical links, where link importance values were derived using the concept of network reliability. A network improvement problem was then solved to minimize the death toll, and a heuristic algorithm was proposed to solve real-sized problems [25]. In particular, there is little related research on emergency logistics network reliability using a complex network [21]. Thus, this research is still unable to provide a theoretical basis and technological instructions for the current construction of emergency logistics.

Using nonlinear dynamics theory and statistics theory, this paper conducts a systematic study of the reliability of emergency logistics networks based on complex network theory. We obtain the changing trends in the reliability index of emergency logistics networks under two modes of attacks through the development of two types of simulated attack procedures, and we verify the complex network characteristics of emergency logistics networks and the effects of different attack modes on its reliability. This work provides a simulation analysis method for emergency network research and a reference for similar research in other fields.

## 2. Complex Emergency Logistics Network Connotation and Its Statistical Characteristics

### 2.1. Topological Structure of Complex Emergency Logistics Network

A complex emergency logistics network can illustrate the interaction and relationships between different nodes within the actual emergency logistics system. These nodes include supply nodes and demand nodes. Emergency materials supply nodes include emergency logistics bases, emergency materials supply bases and emergency allocation centers. The regions are divided into several units, and the center of each unit represents each emergency material’s demand node.

Complex network theory originated from the random graph model initiated by Erdös and Rényi in the 1960s. An increasing number of applications of complex network theory continue to be developed in a variety of fields, including communication, and published in journals such as *Nature* and *Science*. The initiation of small-world networks [26] and scale-free networks [27] was proposed by Watts and Barabasi. Existing research shows that complex networks have characteristics different from statistical features (including the small-world effect and scale-free property [28]) and thus belong to neither random nor regular networks.

**Figure 1:**
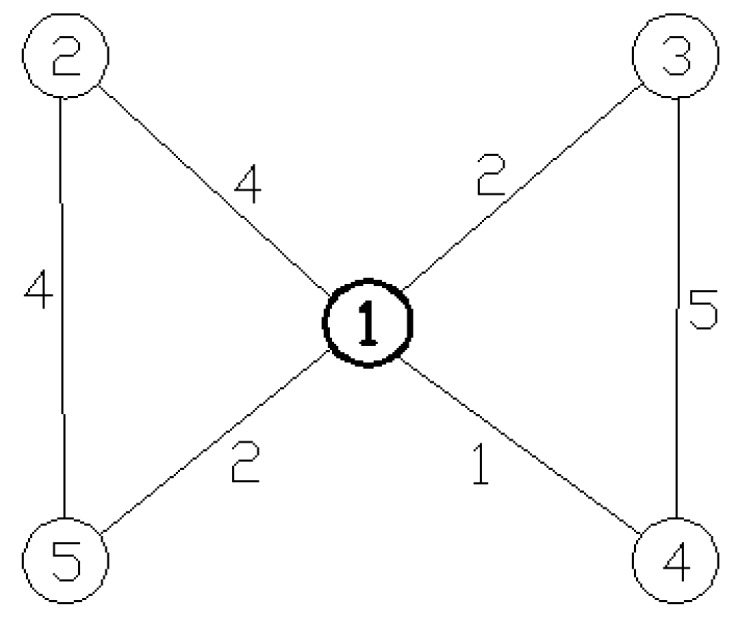
Topological model of emergency logistics network.

Based on the physical structure of an actual emergency logistics network, this paper describes emergency logistics supply nodes, emergency logistics demand nodes, and links between supply and demand nodes in the network. For instance, Figure 1 shows that there are 5 nodes and 6 links in the network. Supply Node 1 directly connects to the rest of the nodes, Supply Node 3 connects to Node 4, and Supply Node 2 connects to Node 5.

Each link between two nodes can be assigned a weight value to indicate specific information about the relationship between nodes, such as in a weighted network, with each side possessing different values to describe the network. The weight of each link is specified according to different research objectives, such as transportation time, actual distance, transportation capacities, and freight traffic volume. For example, Figure 1 shows that it takes 4 h to transport materials from Supply Node 1 to Demand Node 2 and 5 h to transport materials from 3 to 4.

Direct connection refers to a direct connection between two nodes; theoretically, transportation will take an infinite amount of time if there is no connection between two nodes. However, we attempt to take temporary measures to connect a supply node with a demand node, even if there is no connection between them. If the emergency time limit period is L, then the link between two unconnected nodes can be assigned the value L. Nodes of this type cannot be added to the number of effective demand nodes because they cannot be supplied in time.

An adjacency matrix is used to describe the emergency logistics network.

(1) $$D\left(n\right)={\left[d_{ij}\right]}_{n\times n}.$$

If there is a direct connection between nodes i and j, then $d_{ij}$ is the time needed for direct transportation between them; if there is no direct connection between nodes i and j, then $d_{ij}$ is L. n is the total number of nodes in the network.

Thus, a topological model of a complete emergency logistics network consisting of supply nodes, demand nodes, and links was built. The model retains the topological features of the emergency logistics network. Therefore, researchers can study the features of the complex network and judge the network reliability by analyzing its basic geometric features, such as degree distribution, average network path length, and the clustering coefficient.

### 2.2. The Statistical Characteristics of a Complex Emergency Logistics Network

The following are the major parameters used in this paper to describe the abovementioned features [29].

Average path length: The average path length of a complex emergency network is the average value of the path lengths between all node pairs in the network, which describes the degree of separation between nodes in the network. The path length between two nodes is defined as the number of links on the shortest path linking them.

Clustering coefficient C describes the clustering state of nodes of the network. A large C value indicates a tight network and a small C value indicates a loose network. The formula for the clustering coefficient is as follows:

(2) $$C=\frac{1}{n}\overset{n}{\underset{i=1}{\sum }}\frac{2l_{i}}{m_{i}\left(m_{i}-1\right)}$$

where $m_{i}$ is the number of vertices directly connected to vertex $v_{i}$, and $l_{i}$ is the number of links directly connected to vertex $v_{i}$. The network clustering coefficient is the average value of the clustering coefficients of all nodes. 

Complex emergency network node degree k is the total number of links connected to one node.

The complex emergency network degree cumulative probability is the ratio of nodes with node degrees no less than k among all nodes such that

(3) p(k)=n(k)N

where n(k) is the number of nodes with degrees no less than k, and N is the total number of nodes in the network.

Emergency logistics will exhibit the small-world effect if the average path length is small and the clustering coefficient is large; furthermore, the network will exhibit the scale-free property if the relationship between the cumulative probability of one node and that of other nodes fits a power law distribution. 

## 3. Connecting Reliability of Emergency Logistics and Its Evaluation Index

Connection reliability matters in determining whether demand nodes can receive emergency materials from supply nodes when there is an emergency need. Clearly, the reliability is related to the effects introduced by attacks and the topological structure of the network.

The main evaluation indicators used for an emergency logistics network include the following:

(1) Emergency supply time T

The emergency supply time is the amount of time it takes the network to fully supply the necessary emergency materials. This measure is the arithmetic mean value of the supply time of all demand nodes. The supply time of demand nodes refers to the time used to transport emergency materials from supply nodes to each demand node.

(2) Ratio of effective demand nodes P

Effective demand nodes refer to the demand nodes that directly or indirectly connect to the emergency supply nodes in time. The ratio of the effective demand nodes refers to the ratio of the number of effective demand nodes among all demand nodes.

## 4. Simulation Method of Emergency Logistics Network Attack

### 4.1. Attack Types of Emergency Logistics Network

This paper only considers the nodes under attack. Invalidation of one node means the invalidation of all zones connected to this node at the same time. All connections bypassing the node will be cut out. Attacks targeting emergency logistics networks can be divided into random attacks and selective attacks.

(1) Random attacks occur randomly at each node and are typically observed in situations such as natural disasters, accidents, and partial failures.
(2) Selective attacks occur based on the number of direct connections, usually in descending order. These attacks are typically observed in situations such as terrorist attacks and blocking at major nodes.

### 4.2. Simulation Pattern and Method of Random Attack

In the simulated pattern of random attacks, all nodes in the network are attacked randomly, and links connected to the nodes under attack will be invalidated. The minimum transportation time from each supply node to each demand node will be calculated in accordance with the arithmetic of Dijskra. The minimum time will be chosen if one demand node can be supplied by two or more supply nodes. All demand nodes with transportation times less than L are defined as effective demand nodes. We can then calculate the ratio of effective demand nodes of the network P in the rest of the network. The emergency supply time T is the arithmetic mean value of the transportation time of all demand nodes. Then, a node will be randomly chosen for attack, and the ratio of demand nodes P and emergency supply times T for the rest of the network will be calculated. We repeat this process until all nodes are attacked. The following are the steps of the simulation algorithm.

(1) Initialize the adjacency matrix $D\left(n\right)={\left[d_{ij}\right]}_{n\times n}$. If there is a side connecting i, j directly, then $d_{ij}$ is the transportation time between the two nodes. If there is no direct connection between i, j, $d_{ij}$ = L (L is the emergency time limit).
(2) Assume that $r_{1}$ is a member of set [1,n], and $D(r_{1}$,  j) = D(j, $\;r_{1})\;$ = L (j=1,2,…, *n*).
(3) Based on Dijskra, calculate the minimum transportation time of each demand node. The first step is to calculate the minimum transportation time from supply node ps=1 to all demand nodes c[k(m),ps], where ps is the sequence number of supply nodes, m is the number of times needed to obtain the minimum transportation time (nodes marked with P) from supply nodes outward (according to Dijskra), and k(m) is the sequence number of a certain node.
(4) Calculate the minimum transportation time of the next supply node ps=2 to all demand nodes. $c\left[k\left(m_{s}\right),ps\right]$ is the value of supply node $k\left(m_{s}\right)=k\left(m_{t}\right)$. If the value is greater than that of the previous supply node $c\left[k\left(m_{t}\right),ps-1\right]$, then the minimum transportation time is the value calculated in Step (3), and $c\left[k\left(m_{s}\right),ps\right]=c\left[k\left(m_{t}\right),ps-1\right]$.
(5) Repeat Step (4) until all supply nodes and the transportation time of all demand nodes $c\left[k\left(m\right),ps^{*}\right]$ have been calculated, where $ps^{*}$ is the sequence number of the last supply node.
(6) Calculate the emergency supply time T. If the transportation time of one demand node is $c\left[k\left(m\right),ps^{*}\right]$ < L, the node is an effective demand node. Thus, the total number and ratio of effective demand nodes P can be obtained. The arithmetic mean of the transportation time of all demand nodes $c\left[k\left(m\right),ps^{*}\right]$ is the emergency supply time T.
(7) Randomly generate another integer $r_{2}$ in the set [1,n] (except $r_{1}$) and assign $D(r_{2}$, j) = D(j, $r_{2})$ = L.
(8) Return to Step (3) and repeat the process until all integers in the set [1,n] have been used.

### 4.3. Simulation Method of Selective Attacks

Selective attack simulation is generally similar to random attack simulation. However, unlike the simulation of random attacks, this simulation selects the nodes with the highest node degree k as its targets for attack. Consequently, the links connected to the node simultaneously lose their function. The ratio of the effective demand nodes P and emergency supply time T over the rest of the network are then calculated, followed by an attack of the node with the next highest node degree k. Again, the ratio of the effective demand nodes P and emergency supply time T are calculated for the rest of the network. We repeat the process until all nodes in the network are attacked. This simulation only requires changes to Steps (2) and (7) in Section 4.2.

## 5. Case Study of Simulation Analysis

**Figure 2:**
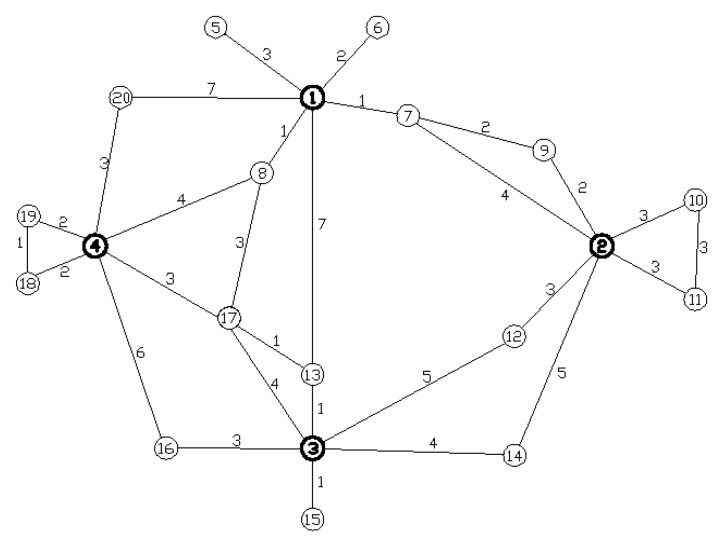
Topological graph of an emergency logistics network in a region.

Figure 2 shows the topological model of an emergency logistics network structure. The model is taken from the eastern coast of China and describes the topological relationships of emergency logistics in the region, assuming that L = 120 h.

Figure 2 clearly shows that there are 20 total nodes in the emergency logistics network in this region. Nodes 1–4, indicated by thick circles, are supply nodes and the rest are demand nodes. There are 29 links, and the minimum transportation time is 1 h. The longest transportation time is 7 h.

### 5.1. Marking of Network Type

To study the characteristics of complex networks, a numerical statement on the degree number k of all nodes and the clustering coefficient C is established according to Figure 2, as shown in Table 1.

Based on the corresponding statistics, there are 12 nodes with node degrees of 1–2, 4 nodes with node degrees of 3–4, and 4 nodes with node degrees exceeding 4, which indicate that the node degrees of most of the nodes are very small and that few nodes have large node degrees.

Additionally, the table shows that the average clustering coefficient C is 0.35, which is far greater than the reciprocal of the nodes 1/20 and that the average path length of the emergency logistics network is 2.758, far smaller than that of the 20 nodes and 29 links of the network. Therefore, this emergency logistics network is a small-world network because of its relatively large clustering coefficient and small average path length.

**Figure 3:**
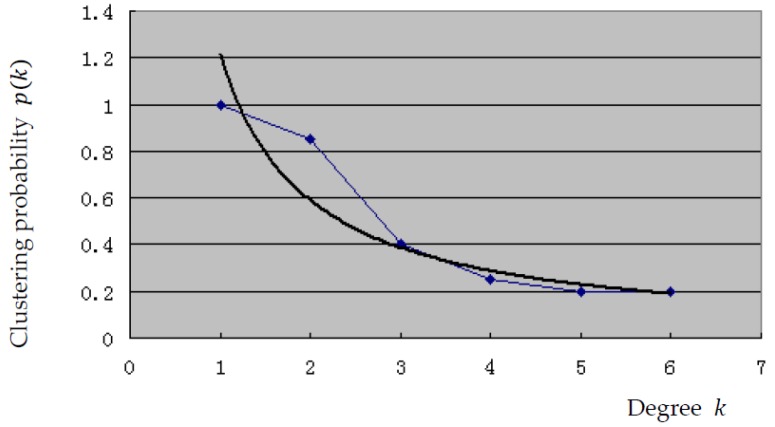
Relation between degree k and its cumulative probability p(k).

The characteristics of the scale-free property of the network are discussed next. In Figure 3, the broken line shows the relationship between the degree of this network and the cumulative probability while the curve is generated by fitting a power function. The curvilinear equation is $p\left(k\right)=1.2124k^{-1.0396}$, and the coefficient of determination is $R^{2}=0.9183$. Therefore, the cumulative degree distribution of the network is consistent with the power law distribution of the dispersion index λ=1.0396. This finding indicates that this emergency logistics network possesses the scale-free property according to complex network theory.

The abovementioned findings demonstrate that this emergency logistics network is a small-world and a scale-free network.

### 5.2. Simulation Results and Its Analysis

**Figure 4:**
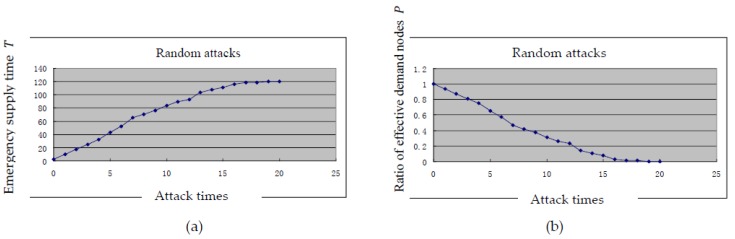

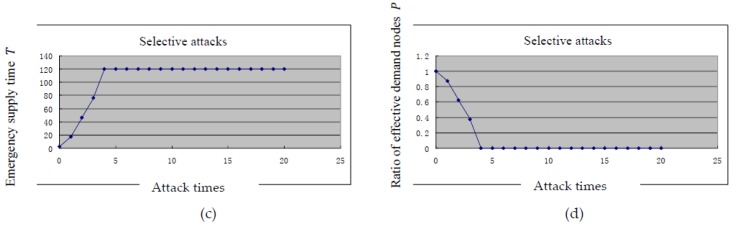
Simulation results under two modes of attack.

To save the distribution time and reduce environmental and public health losses in post-accident operations and emergency rescue, based on the simulation method outlined above, we developed a simulation model using the Visual Basic platform to analyze random and selective attacks on the emergency logistics network. The changing state of the emergency supply time T and the ratio of effective demand nodes P under random attacks are shown in Figure 4a,b. The changing state of the emergency supply time T and the ratio of effective demand nodes P under selective attacks are shown in Figure 4c,d.

The simulation results indicate that the supply time is T = 2.25 h and the effective demand ratio is P = 1 in the initial state of the emergency logistics network when there are no attacks. When facing random attacks, supply time T and effective demand node P change steadily. There is an approximately linear relationship between the number of attacks and these two respective measures, and they reach their final values after the final attack (supply time T = 120 h, effective demand ratio P = 0). This finding indicates that the topological structure of the emergency logistics network does not change suddenly and that this network enjoys a high degree of reliability. This observation is determined by the scale-free property of the network. To be exact, nodes under random attacks usually correspond to demand nodes, and the number of demand nodes with small connecting degrees is far greater than the number of supply nodes with large connecting degrees. The non-functionality of these demand nodes will not introduce severe consequences because they are not required to send supplies to other nodes and have few connections.

Figure 4 shows that the ratio of effective demand nodes is on average less than 0.5 after 7 random attacks or 3 selective attacks, which indicates that the emergency logistics network is very fragile when facing selective attacks. Selective attacks can damage vulnerable supply nodes with large network connectivity, which will severely affect the reliability of the emergency logistics network. The abovementioned analysis suggests that selective attacks will greatly increase the transportation time T of the emergency logistics network and significantly decrease the ratio of demand nodes P. After four attacks, the two will reach their final values, which indicates that the network will have lost its functionality and will no longer be able to fulfill any emergency logistics tasks. Therefore, to achieve efficient operation of the network, several suggestions are put forward:

(1) Special attention should be paid to the protection of supply nodes and nodes with high connectivity, such as emergency logistics conversion nodes. A dynamic, flat emergency supplies reserve mechanism and network should be established. The market-oriented storage and government reserves should be combined with the integration of the central and local emergency supply nodes to achieve the linkage between the reserve nodes.
(2) We should accelerate the construction of an emergency logistics channel so that we can find an alternate link when one link is blocked in post-accident rescue. After the timeliness and safety of transportation routes are focused on, several alternative transportation plan should be prepared in advance, the corresponding alternative transportation plan will be immediately activated in post-accident rescue.

## 6. Conclusions 

This paper applies complex network theory to emergency logistics research. We analyzed statistical characteristics of complex emergency logistics networks and defined their connected reliability and evaluation indicators. Then, a simulation model was established to analyze the reliability of an emergency logistics network under two modes of attack. The simulation method provides references for emergency logistics network reliability research and evaluation. Although complex network theory is a hot research topic, its application to logistics, particularly emergency logistics, has not been reported. Thus, introducing this theory into emergency logistics studies may provide a new research tool for this field and reduce environmental and public health losses in post-accident operations and emergency rescues.

This paper assumes that the distribution of emergency materials can be completed as long as there are connections between supply nodes and demand nodes. This paper does not consider the demand and supply of emergency materials, which is a simplification of real-world conditions. Therefore, we will take the actual transportation volume into consideration in future emergency logistics reliability research. In addition, various data on emergency logistics networks can now be collected. It would be worthwhile to further investigate the sustainability of complex emergency logistics networks under recent diseases or public health emergencies in China based on data-driven quantitative analysis.

## Figures and Tables

**Table 1:** Degree number and clustering coefficient of all nodes.

Node Number	Degree k	Clustering Coefficient C	Node Number	Degree k	Clustering Coefficient C
1	6	0	11	2	1
2	6	0.13	12	2	0
3	6	0.67	13	3	0.33
4	6	0.13	14	2	0
5	1	0	15	1	0
6	1	0	16	2	0
7	3	0.33	17	4	0.33
8	3	0.33	18	2	1
9	2	1	19	2	1
10	2	1	20	2	0
